# Supplementary material for: The AMPK-Sirtuin 1-YAP axis is regulated by fluid flow intensity and controls autophagy flux in kidney epithelial cells
Source: Nat Commun. 2023 Dec 5;14:8056. doi: 10.1038/s41467-023-43775-1 (PMC10698145; doi:10.1038/s41467-023-43775-1)
Supplement: Supplementary file 1 — Supplementary Information [file 41467_2023_43775_MOESM1_ESM.pdf]

Supplementary information

The AMPK-Sirtuin 1-YAP axis is regulated by fluid flow intensity and controls autophagy flux in kidney epithelial cells.

Aurore Claude-Taupin[1*], Pierre Isnard[1], Alessia Bagattin[1], Nicolas Kuperwasser[2], Federica Roccio[1], Biagina Ruscica[1], Nicolas Goudin[2], Meriem Garfa-Traoré[2], Alice Regnier[1], Lisa Turinsky[1], Martine Burtin[1], Marc Foretz[3], Marco Pontoglio[1], Etienne Morel[1], Benoit Viollet[3], Fabiola Terzi[1#], Patrice Codogno[1#] and Nicolas Dupont[1*#].

[1] Université Paris Cité, INSERM UMR-S1151, CNRS UMR-S8253, Institut Necker Enfants Malades, F-75015 Paris, France.
[2] Structure Fédérative de Recherche Necker, US24-UMS3633, Paris, France.
[3] Institut Cochin, Inserm U1016 - CNRS UMR8104 – Université Paris Cité, 75014, Paris, France

[#] These authors jointly supervised this work
* Co-corresponding authors:

aurore.claude-taupin@inserm.fr
nicolas.dupont@inserm.fr

Supplementary Tables

Supplementary Table 1: Patient characteristics.

Characteristic	Control	DKD
Sex – number (%)		
Male	3 (50%)	4 (66.7%)
Female	3 (50%)	2 (33.3%)
Age – mean ± SD	45.8 ± 20.5	57.3 ± 13.7
Age - min, median, max	14, 50, 71	42, 57, 75

Supplementary Table 2: List of siRNAs used in this study.

Target cells	siRNA name	Qiagen Cat #	Target sequence
KECs	Mm_Yap1_6	SI02689113	ACCCTTGAACATATACATTTA
	Mm_Yap1_7	SI02711205	AACATCCTATTTAAATCTTAA
	Mm_Wwtr1_6	SI02697149	CTGCATTTCTGTGGCAGATAA
	Mm_Wwtr1_7	SI02720599	TTCCTTAATCACATAGAGAAA
	Mm_Kif3a_2	SI00175987	ACGAACCTCCAAAGACATTTA
	Mm_Kif3a_4	SI00176001	GACCCAGAGGTTAGAGGTTAA
	Mm_Lkb1(Stk11)_1	SI01435735	CAGGGCGGTCAAGATCCTCAA
	Mm_Lkb1(Stk11)_2	SI01435742	CCGAGGGATGTTGGAGTATGA
	Mm_Lats1_1	SI02766687	CAGGAAATGTGCAACATTCAA
	Mm_Lats1_4	SI02774821	TAGTGTATGTTTAATAAACTA
	Mm_Lats2_7	SI04941636	CCCGAAGTTTGGACCTTATCA
	Mm_Lats2_8	SI04941643	GAAGATTGTATTTATGGTAAA
	Mm_Cep164_1	SI00964663	CGCGACCATGAAAGGAAATTA
	Mm_Cep164_4	SI00964684	CTTGATATTTCTCTTCTTTAA
	Mm_Atg5_6	SI02720186	ACAGTTTGTATTTCTGATTAA
	Mm_Atg5_7	SI02745435	AAGGAAGAACTTAGCCTATAT
HK-2	Hs_YAP1_6	SI04438637	CACATCGATCAGACAACAACA
	Hs_YAP1_7	SI04438651	CCGGGATGTCTCAGGAATTGA
	Hs_WWTR1_2	SI00111223	CTGGCTGTAATCACTACCATT
	Hs_WWTR1_3	SI00111230	AGACATGAGATCCATCACTAA

Supplementary Table 3: List of plasmids used in this study.

Plasmid	Source	Identifier
pCMV-FLAG-YAP-5SA	Addgene	#27371
pCMV-FLAG-YAP-5SA/S94A	Addgene	#33103
pDONR221-P5P2-FLAG-YAP1	Addgene	#79503
pDONR221-P5P2-FLAG-YAP1-S61A	This study	
pCMV-mRFP-FLAG-YAP1	This study	
pCMV-mRFP-FLAG-YAP1-S61A	This study	
pFLAG-mTAZ	Dupont S.	PMID: 21654799
pFLAG-mTAZ-4SA	Dupont S.	PMID: 21654799
8xGTIIC-luciferase	Addgene	#34615
pRL-TK-Renilla-Luciferase	Promega	#E2241
pInducer20 EGFP-TEADi	Addgene	#140145
mRFP-EGFP-LC3 (ptfLC3)	Addgene	#21074
pCas9-sgYAP	This study	
pEGFP	Addgene	#31796
pLentiGFP-YAP(WT)	This study	
pLentiGFP-YAP(5SA)	This study	
pLentiGFP-YAP(5SA/S94A)	This study	
pLentiGFP-YAP(S61A)	This study	
pSIRT1-WT-EGFP	Horio S.	PMID: 17197703
pSIRT1-NLSmut-EGFP	Horio S.	PMID: 17197703
pSIRT1-NESmut-EGFP	Horio S.	PMID: 17197703

Supplementary Table 4: List of qRT-PCR primers (mRNA) used in this study.

Target	Forward	Reverse
Ankrd1	CTGTGAGGCTGAACCGCTAT	CCAGTGCAACACCAGATCCA
β-actin	GGCCAACCGTGAAAAGATGA	ACCAGAGGCATACAGGGACAG
Bcl2	GTGGATGACTGAGTACCT	CCAGGAGAAATCAAACAGAG
Cptp	GTGGAAGGAAACTAGGCCCC	CCAGTGGAAAAGCGTAGGGT
Cyr61	AGAGGCTTCCTGTCTTTGGC	CCAAGACGTGGTCTGAACGA
Ptplad2	GTCGAACAGAGCAGGAGGAAAC	CATTTGCTGTTGCCCAAGGAAT
Ppargc1a	TAAACTGAGCTACCCTTGG	CTCGACACGGAGAGTTAAAGGAA
Rubicon	CTCTGAGCAAGACTTTGGCAGC	GCACTTCATCAGCTCAATGGCG
Tfam	CCGAAGTGTTTTTCCAGCAT	GCGTGCAATTTTCCTAACCA
Last1	TGGGACAACCTCCTTTCTTGGC	TGAGGTCAGAGGCTTCAGGACT
Lats2	GCACTGGATTCAGGTGGACTCA	CGACAGTTGGAAACATCGTCCC

Supplementary Table 5: List of qRT-PCR primers (ChIP) used in this study.

Target	Forward	Reverse
RUBCN	TCCTAAAATGCCCCCAGAAC	GCCTCTGGAATGGCAGTTTC
RUBCN -1000	AACCCATCTCCGCCTGAGTC	GGGACTGGCTTTACTGTGAGC
RUBCN -3500	TATCACCGGCATGTTGACCC	TGAGCCTGAATCCCTGTGTG
RUBCN +900	GTGCAGTTGAAGGCAGGAGC	CTGCTCTCCTCGTCCTGTGG
RUBCN +2400	CATGTCCCTGCACAGCCG	GCCAACTGCCCCAATGTCTG
CTGF	GTGGAGTGTCAAGGGGTCAG	CACATTCCTCCCCACCTTCC
CTGF -1600	AGCCACTCACACCTTTCGCT	ACCTTGTTAAGCCAGCCACA
CTGF -2700	ACCCTTCTCCTTCTGTCCCA	AGACAGAAGACTACCTCTCCACA
CTGF +700	AGAACTGTGTACGGAGCGTG	CCAGTGCACACTCCGATCTT
CTGF +3000	TGCAGTGGGAATTGTGACCT	AGCTTTATCACCTGCACAGC

Supplementary Figures

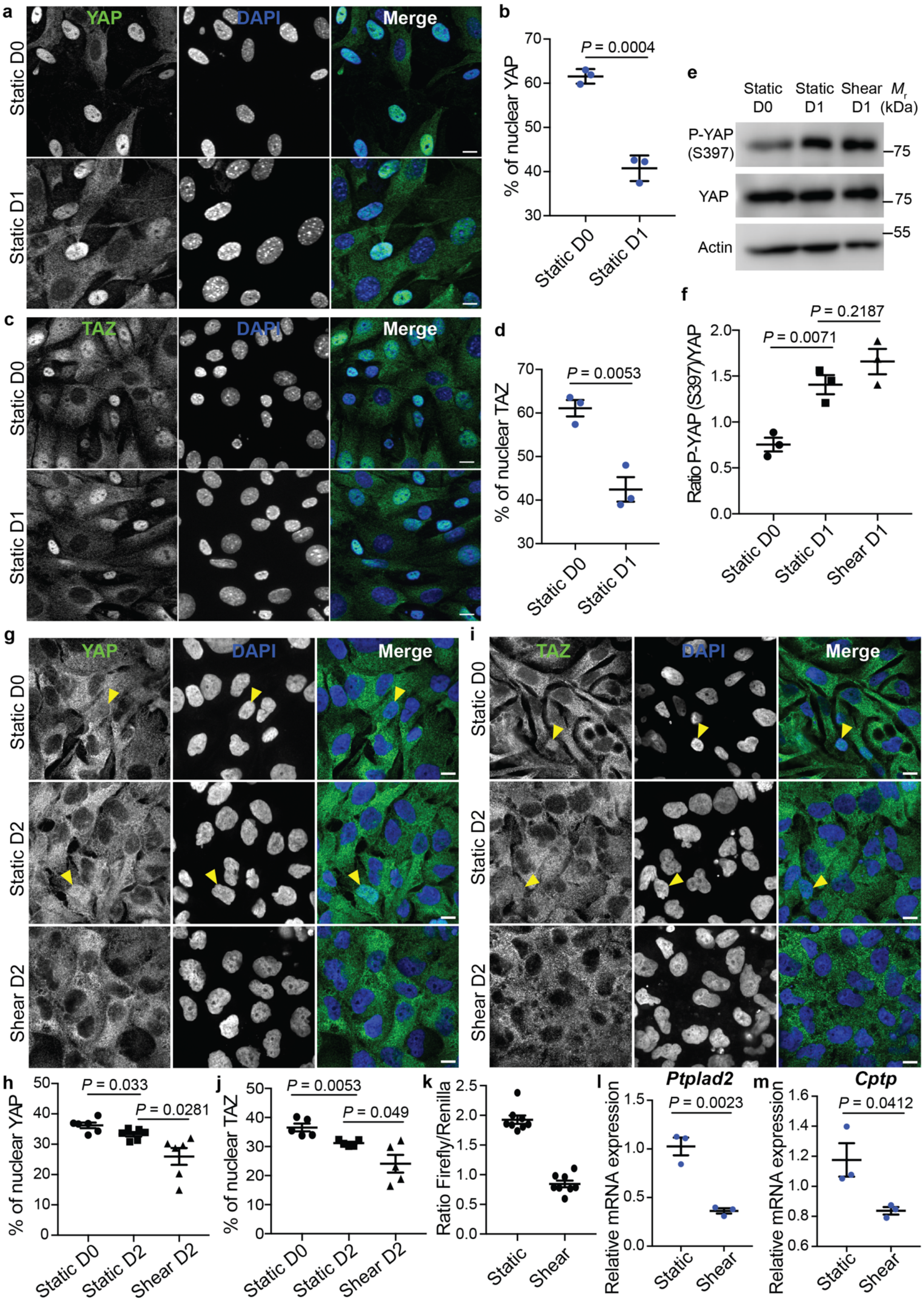

a
YAP
DAPI
Merge
Static D0
Static D1
b
P = 0.0004
% of nuclear YAP
70
60
50
40
30
Static D0
Static D1
e
Static D0
Static D1
Shear D1
M_r (kDa)
P-YAP (S397)
YAP
Actin
75
75
55
c
TAZ
DAPI
Merge
Static D0
Static D1
d
P = 0.0053
% of nuclear TAZ
Static D0
Static D1
f
P = 0.2187
P = 0.0071
Ratio P-YAP (S397)/YAP
2.0
1.5
1.0
0.5
0.0
Static D0
Static D1
Shear D1
g
YAP
DAPI
Merge
Static D0
Static D2
Shear D2
i
TAZ
DAPI
Merge
Static D0
Static D2
Shear D2
h
P = 0.033
P = 0.0281
% of nuclear YAP
Static D0
Static D2
Shear D2
j
P = 0.0053
P = 0.049
% of nuclear TAZ
Static D0
Static D2
Shear D2
k
Ratio Firefly/Renilla
Static
Shear
l
Ptplad2
P = 0.0023
Relative mRNA expression
Static
Shear
m
Cptp
P = 0.0412
Relative mRNA expression
Static
Shear

Supplementary Figure 1: YAP/TAZ inhibition by shear stress leads to the downregulation of flow-specific YAP/TAZ target genes.

(a,b) Representative images **(a)** and quantification **(b)** of YAP nuclear localization in KECs (Static D0) or kept in culture for 24h (Static D1). Data show the mean ± s.e.m.; n = 3 independent experiments, two-sided *t*-test. Scale bars, 10 μm. **(c,d)** Representative images **(c)** and quantification **(d)** of TAZ nuclear localization in KECs (Static D0) or kept in culture for 24h (Static D1). Data show the mean ± s.e.m.; n = 3 independent experiments, two-sided *t*-test. Scale bars, 10 μm. **(e)** Representative images of P-YAP (S397), YAP and Actin proteins levels in KECs subjected to flow (shear) or not (static) during 24h, by western blot analysis. **(f)** The ratio of P-YAP (S397) to YAP was determined by densitometry, relative to panel e. Data show the mean ± s.e.m.; n = 3 independent experiments, two-sided *t*-test. **(g,h)** Representative images **(g)** and quantification **(h)** of YAP nuclear localization in HK-2 subjected to flow (shear) or not (Static D0 and Static D2). For the “Static D2” condition, cells are kept in culture for 48h. Data show the mean ± s.e.m.; n = 6 from 3 independent experiments, two-sided *t*-test. Scale bars, 10 μm. **(i,j)** Representative images **(i)** and quantification **(j)** of TAZ nuclear localization in HK-2 subjected flow (shear) or not (Static D0 and Static D2). For the “Static D2” condition, cells are kept in culture for 48h. Data show the mean ± s.e.m.; n = 6 from 3 independent experiments, two-sided *t*-test. Scale bars, 10 μm. **(k)** Luciferase assay for YAP/TAZ activity in HK-2 subjected to shear stress or not (static) during 48h. Data show the mean ± s.e.m.; n = 8 from 3 independent experiments, two-sided *t*-test. **(l,m)** Expression of *Ptplad2* **(l)** and *Cptp* **(m)** in KECs subjected to flow (shear) or not (static) during 24h. mRNA levels were quantified by real-time RT-qPCR, normalized to β-actin

and are presented as fold increases. Data show the mean ± s.e.m.; n = 3 independent experiments, two-sided *t*-test. Source data are provided as a Source Data file.

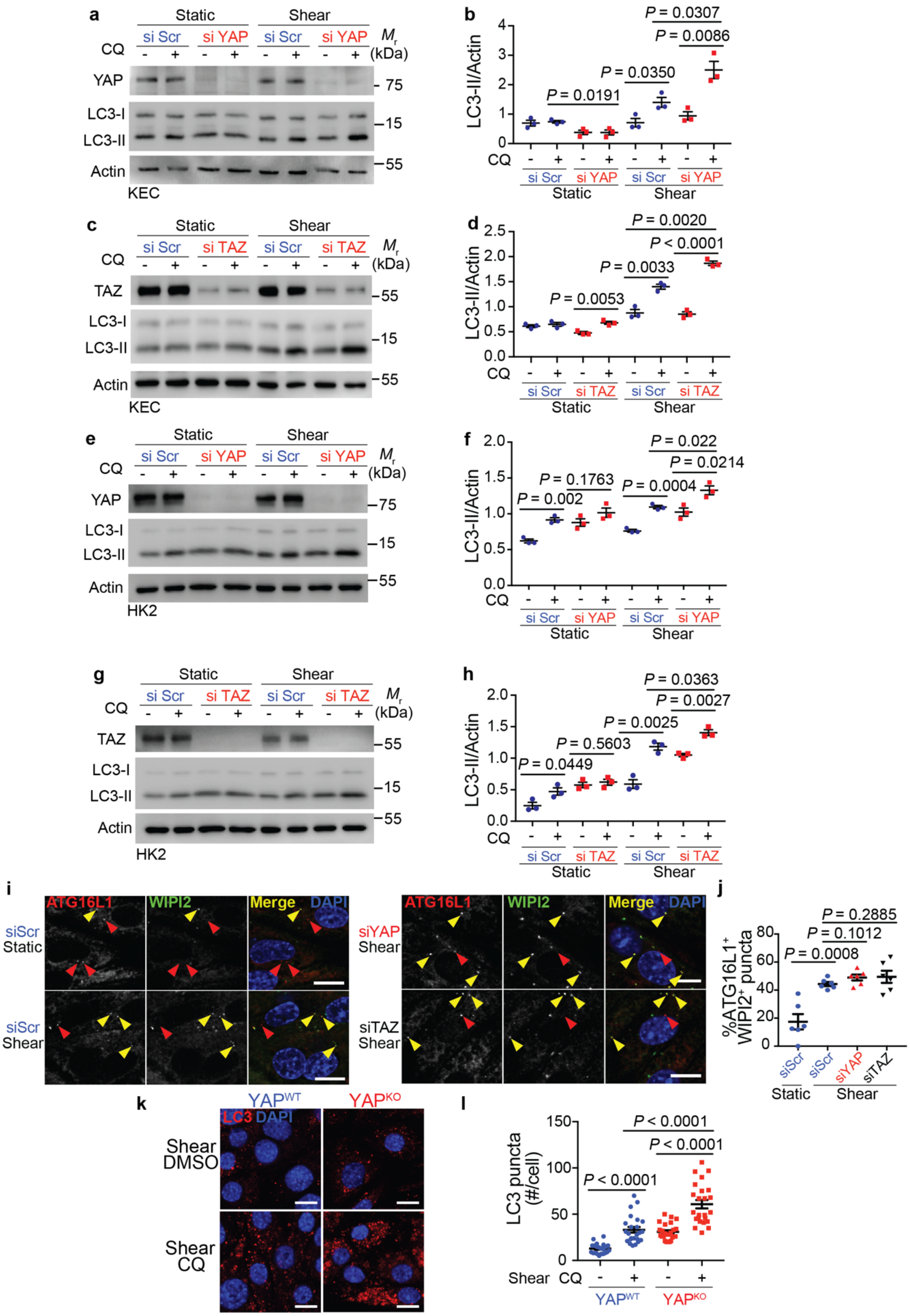

a
Static
Shear
si Scr
si YAP
CQ
M_r (kDa)
YAP
LC3-I
LC3-II
Actin
75
15
55
KEC
b
LC3-II/Actin
P = 0.0307
P = 0.0086
P = 0.0350
P = 0.0191
c
si TAZ
TAZ
d
P = 0.0020
P < 0.0001
P = 0.0033
P = 0.0053
e
HK2
f
P = 0.022
P = 0.0214
P = 0.1763
P = 0.0004
P = 0.002
g
h
P = 0.0363
P = 0.0027
P = 0.0025
P = 0.5603
P = 0.0449
i
ATG16L1
WIPI2
Merge
DAPI
siScr
siYAP
siTAZ
j
%ATG16L1+ WIPI2+ puncta
P = 0.2885
P = 0.1012
P = 0.0008
k
YAP^WT
YAP^KO
LC3
Shear DMSO
Shear CQ
l
LC3 puncta (#/cell)
P < 0.0001
P < 0.0001
P < 0.0001

Supplementary Figure 2: The loss of YAP or TAZ protein stimulates autophagy flux during shear stress.

(a) Representative images of YAP, LC3-I, LC3-II and Actin proteins levels in KECs after transfection with a control siRNA (si Scr) or siRNA against *YAP*, subjected to flow (shear, 4 days) or not (static), in the presence or absence of chloroquine (CQ), by western blot (WB). **(b)** The ratio of LC3-II to Actin was determined by densitometry, relative to panel a. **(c)** Representative images of TAZ, LC3-I, LC3-II and Actin proteins levels in KECs after transfection with a control siRNA (si Scr) or siRNA against *TAZ*, subjected to flow (shear, 4 days) or not (static), in the presence or absence of chloroquine (CQ), by WB. **(d)** The ratio of LC3-II to Actin was determined by densitometry, relative to panel c. **(e)** Representative images of YAP, LC3-I, LC3-II and Actin proteins levels in HK-2 after transfection with a control siRNA (si Scr) or siRNA against *YAP*, subjected to flow (shear, 2 days) or not (static), in the presence or absence of chloroquine (CQ), by WB. **(f)** The ratio of LC3-II to Actin was determined by densitometry, relative to panel e. **(g)** Representative images of TAZ, LC3-I, LC3-II and Actin proteins levels in HK-2 after transfection with a control siRNA (si Scr) or siRNA against *TAZ*, subjected to flow (shear, 2 days) or not (static), in the presence or absence of chloroquine (CQ), by WB. **(h)** The ratio of LC3-II to Actin was determined by densitometry, relative to panel g. **(i,j)** Representative images **(i)** and quantification **(j)** of colocalization between ATG16L1 and WIPI2 in KECs after transfection with a control siRNA (si Scr) or siRNA against *YAP or TAZ*, subjected to flow (shear, 1 day) or not (static). **(k,l)** Representative images **(k)** and quantification **(l)** of LC3 puncta in YAP^{WT} and YAP^{KO} KECs subjected to flow (shear, 1 day) or not (static) during 24h in the presence or absence of chloroquine (CQ). **(b,d,f,h)**

Data show the mean ± s.e.m.; n = 3 independent experiments, two-sided *t*-test. **(j,l)** Data show the mean ± s.e.m. n = 6 (for **j**) or 24 (for **l**) from 3 independent experiments, two-sided *t*-test. Scale bars, 10 μm. Source data are provided as a Source Data file.

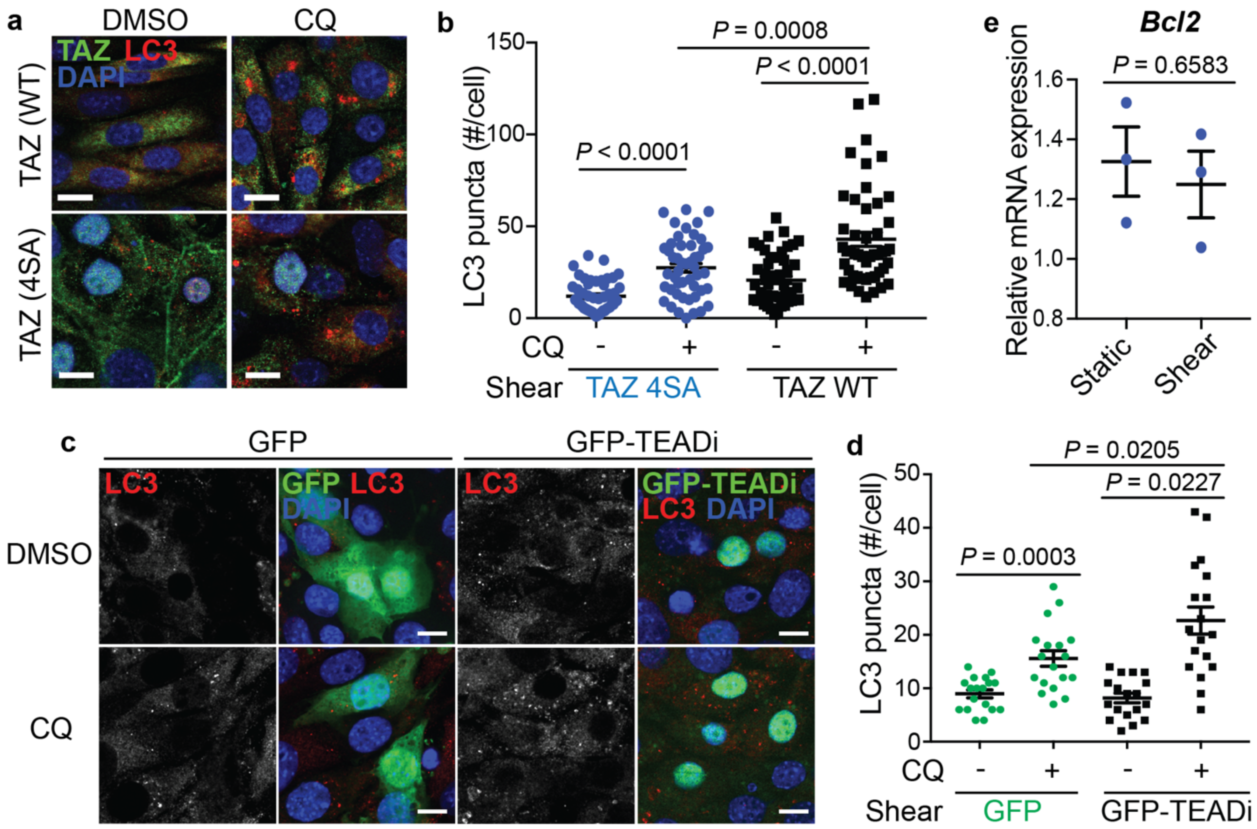

Supplementary Figure 3: The expression of a constitutively inactive form of TAZ or inhibition of YAP/TAZ interaction with TEAD transcription factors stimulate the autophagy flux during shear stress.

(a,b) Representative images **(a)** and quantification **(b)** of LC3 puncta in KECs transfected with TAZ WT or its constitutively active form (TAZ 4SA), subjected to shear stress during 24h in the presence or absence of chloroquine (CQ). Data show the mean ± s.e.m.; n = 48 individual data points from 3 independent experiments, two-sided *t*-test. **(c,d)** Representative images **(c)** and quantification **(d)** of LC3 puncta in GFP or GFP-TEADi-transfected cells subjected to flow (shear) or not (static) during 24h in the presence or absence of chloroquine (CQ). Data show the mean ± s.e.m.; n = 18 from 3 independent experiments, two-sided *t*-test. Scale bars, 10 μm. **(e)** Expression of *Bcl2* in KECs subjected to flow (shear) or not (static) during 24h. mRNA levels were quantified by real-

time RT-qPCR, normalized to β-actin and are presented as fold increases. Data show the mean ± s.e.m.; n = 3 independent experiments, two-sided *t*-test. Source data are provided as a Source Data file.

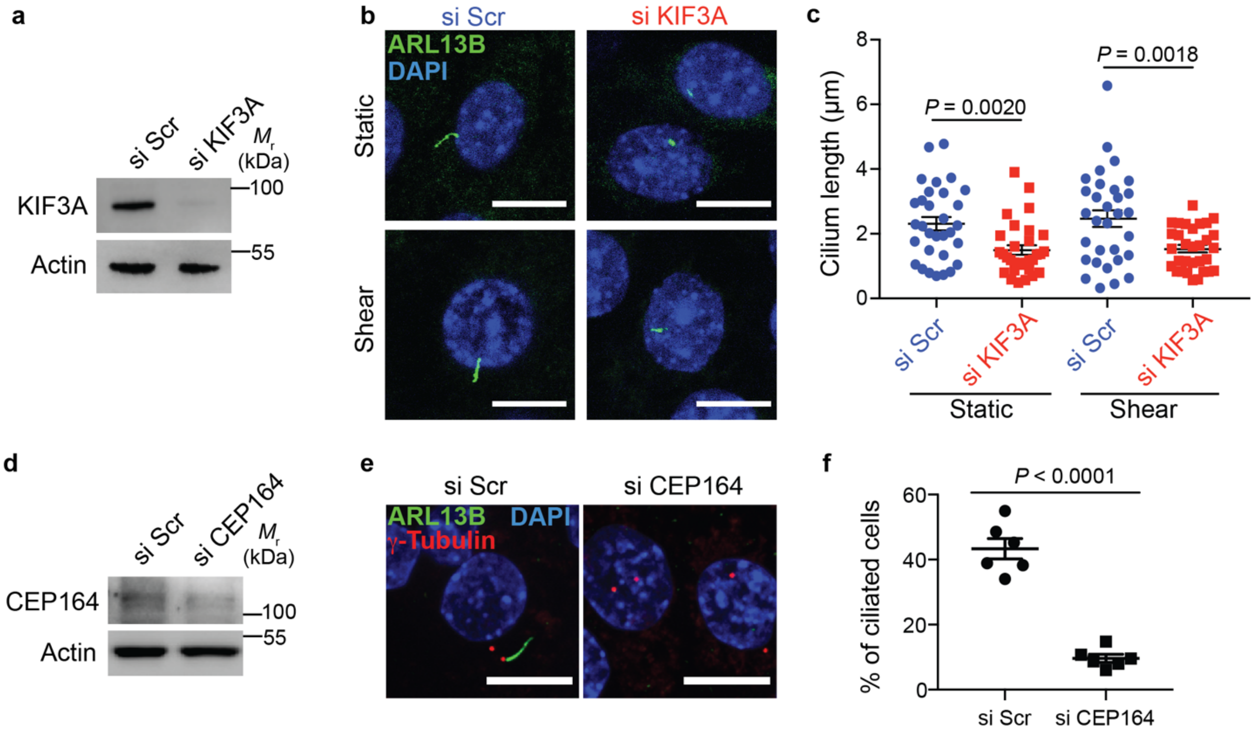

Supplementary Figure 4: KIF3A and CEP164 downregulation in KECs affects ciliogenesis.

(a) Confirmation by immunoblotting of KIF3A knockdown in KEC cells. Representative images from n=3 independent experiments shown. **(b,c)** Representative images **(b)** and quantification **(c)** of cilia length (ARL13B$^+$) in KECs after transfection with a control siRNA (si Scr) or siRNA against *KIF3A*, subjected to flow (shear) or not (static) during 24h. Data show the mean ± s.e.m.; n = 31 individual data points from 3 independent experiments, two-sided *t*-test. Scale bars, 10 µm. **(d)** Confirmation by immunoblotting of CEP164 knockdown in KEC cells. Representative images from n=3 independent experiments shown. **(e,f)** Representative images **(e)** and quantification **(f)** of cilia number (ARL13B$^+$, γ-tubulin$^+$) in KECs after transfection with a control siRNA (si Scr) or siRNA against *CEP164*, subjected to flow (shear) or not (static) during 24h. Data show the mean ±

s.e.m.; n = 6 individual data points from 3 independent experiments, two-sided *t*-test. Scale bars, 10 µm. Source data are provided as a Source Data file.

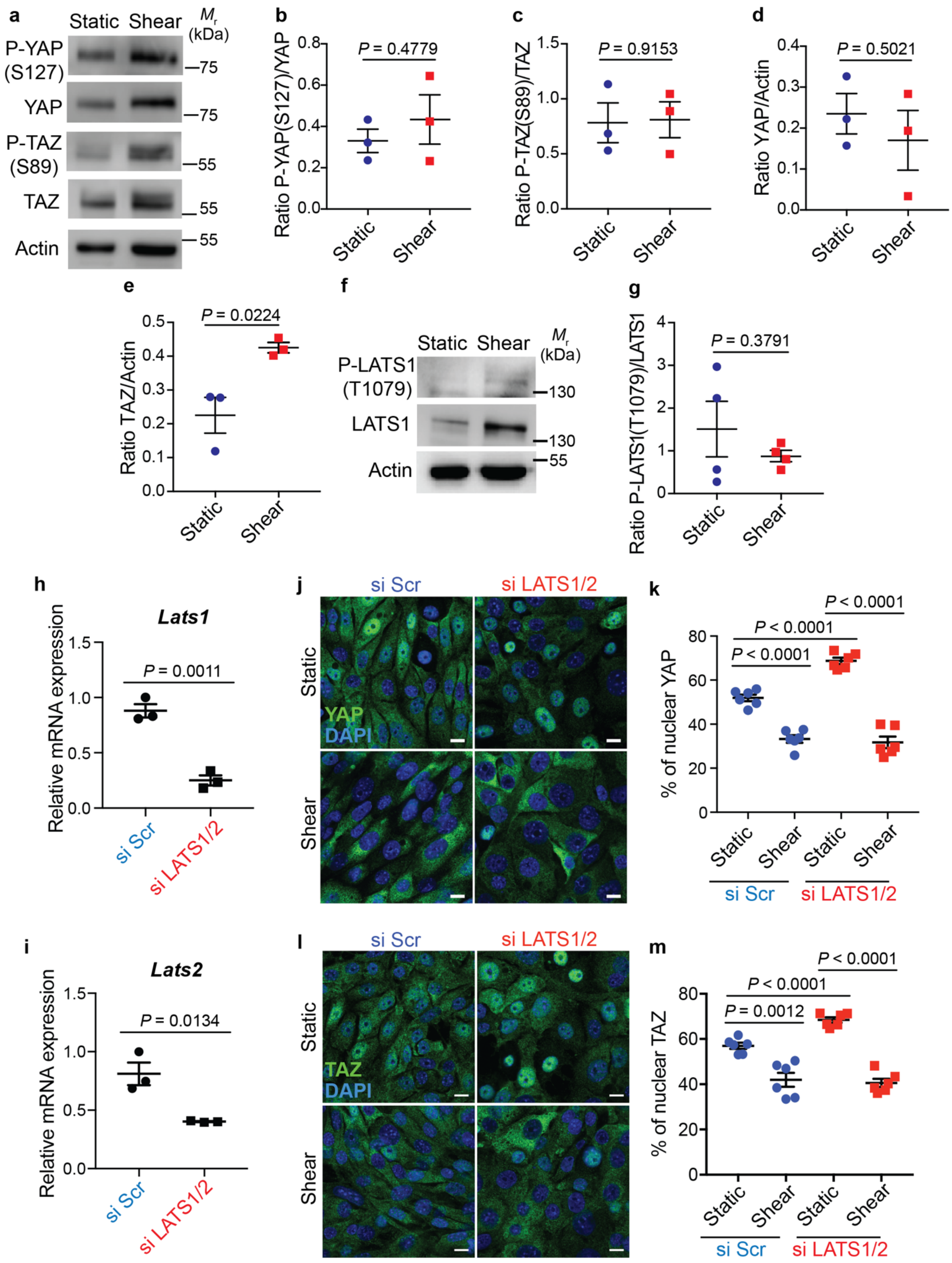

a
Static
Shear
M_r (kDa)
P-YAP (S127)
YAP
P-TAZ (S89)
TAZ
Actin
75
75
55
55
55
b
Ratio P-YAP(S127)/YAP
P = 0.4779
c
Ratio P-TAZ(S89)/TAZ
P = 0.9153
d
Ratio YAP/Actin
P = 0.5021
e
Ratio TAZ/Actin
P = 0.0224
f
P-LATS1 (T1079)
LATS1
Actin
130
130
55
g
Ratio P-LATS1(T1079)/LATS1
P = 0.3791
h
Lats1
Relative mRNA expression
P = 0.0011
si Scr
si LATS1/2
i
Lats2
P = 0.0134
j
si Scr
si LATS1/2
Static
Shear
YAP
DAPI
k
% of nuclear YAP
P < 0.0001
P < 0.0001
P < 0.0001
l
TAZ
DAPI
m
% of nuclear TAZ
P = 0.0012
P < 0.0001
P < 0.0001

Supplementary Figure 5: YAP/TAZ inactivation during shear stress is Hippo independent.

(a) Representative images of P-YAP (S127), YAP, P-TAZ (S89), TAZ and Actin proteins levels in KECs subjected to flow (shear) or not (static) during 24h, by western blot analysis. **(b-e)** The ratio of P-YAP to YAP **(b)** P-TAZ to TAZ **(c)** YAP to Actin **(d)** and TAZ/Actin **(e)** was determined by densitometry, relative to panel a. Data show the mean ± s.e.m.; n = 3 independent experiments, two-sided *t*-test. **(f)** Representative images of P-LATS1 (T1079), LATS1 and Actin proteins levels in KECs subjected to flow (shear) or not (static) during 24h, by western blot analysis. **(g)** The ratio of P-LATS1 to LATS1 was determined by densitometry, relative to panel f. Data show the mean ± s.e.m.; n = 3 independent experiments, two-sided *t*-test. **(h,i)** Confirmation by real-time RT-qPCR of *LATS1* and *LATS2* knockdown in KEC cells. mRNA levels were quantified by real-time RT-qPCR, normalized to β-actin and are presented as fold increases. Data show the mean ± s.e.m.; n = 3 independent experiments, two-sided *t*-test. **(j,k)** Representative images **(j)** and quantification **(k)** of YAP nuclear localization in KECs after transfection with a control siRNA (si Scr) or siRNA against *LATS1 and LATS2*, subjected to flow (shear) or not (static) during 24h. Data show the mean ± s.e.m.; n = 6 from 3 independent experiments, two-sided *t*-test. Scale bars, 10 µm. **(l,m)** Representative images **(l)** and quantification **(m)** of TAZ nuclear localization in KECs after transfection with a control siRNA (si Scr) or siRNA against *LATS1 and LATS2*, subjected to flow (shear) or not (static) during 24h. Data show the mean ± s.e.m.; n = 6 from 3 independent experiments, two-sided *t*-test. Scale bars, 10 µm. Source data are provided as a Source Data file.

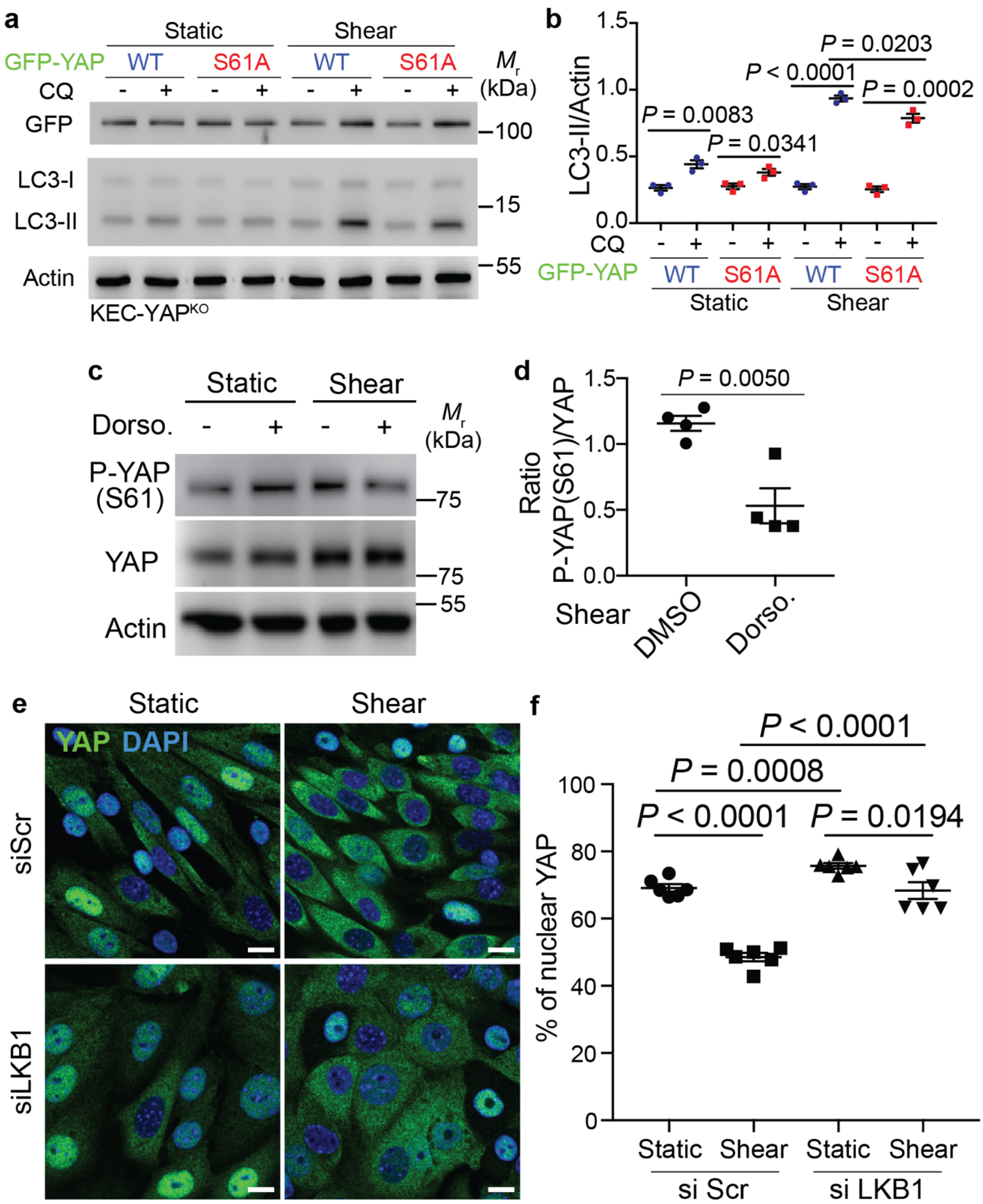

Supplementary Figure 6: An AMPK-dependent phosphorylation of YAP at S61 regulates autophagy upon fluid flow.

(a) Representative images of GFP, LC3-I, LC3-II and Actin proteins levels in KECs expressing a wild-type (WT) or a mutant (S61A) form of YAP, then subjected to flow (shear, during 1 day) or not (static), in the presence or absence of chloroquine (CQ), by western blot analysis. **(b)** The ratio of LC3-II to Actin was determined by densitometry, relative to panel a. Data show the mean ± s.e.m.; n = 3 independent experiments, two-sided *t*-test**.** **(c)** Representative images of P-YAP (S61), YAP and Actin proteins levels in KECs subjected to flow (shear) or not (static) during 24h, in the presence or absence of dorsomorphin (Dorso.) by western blot analysis. **(d)** The ratio of P-YAP (S61) to YAP was determined by densitometry, relative to panel c. Data show the mean ± s.e.m.; n = 4 independent experiments, two-sided *t*-test**.** **(e,f)** Representative images **(e)** and quantification **(f)** of YAP nuclear localization in KECs after transfection with a control siRNA (si Scr) or siRNA against *LKB1*, subjected to flow (shear) or not (static) during 24h. Data show the mean ± s.e.m.; n = 6 from 3 independent experiments, two-sided *t*-test. Scale bars, 10 µm. Source data are provided as a Source Data file.

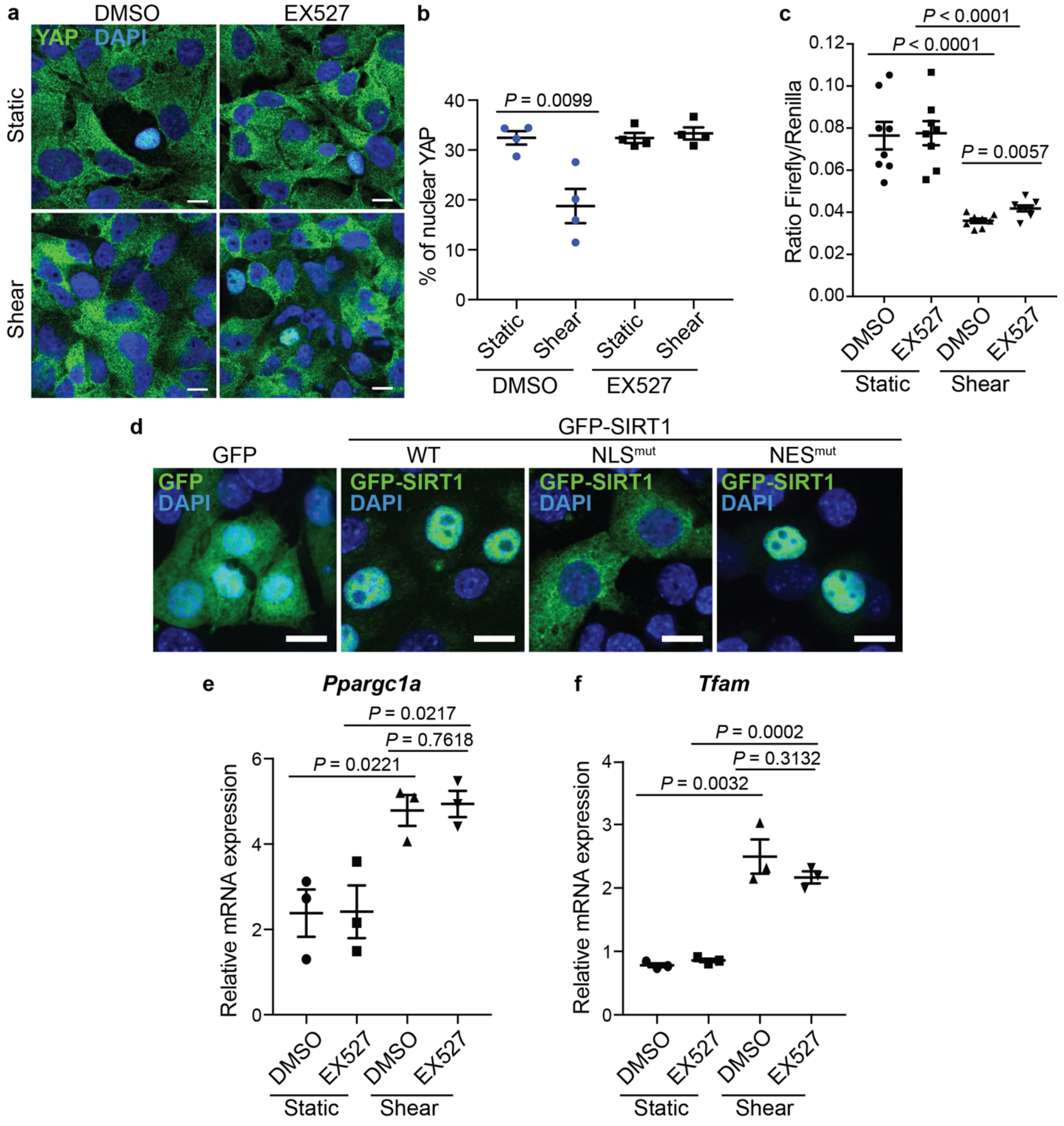

Supplementary Figure 7: YAP cytoplasmic retention, dependent on SIRT1, does not influence mitochondrial biogenesis.

(a,b) Representative images **(a)** and quantification **(b)** of YAP nuclear localization in HK-2 treated or not with EX527, subjected to flow (shear) or not (static) during 24h. Data show the mean ± s.e.m.; n = 4 independent experiments, two-sided *t*-test. Scale bars, 10

µm **(c)** Luciferase assay for YAP/TAZ activity in KECs subjected to shear stress or not (static) during 24h, in the presence or absence of EX527. Data show the mean ± s.e.m.; n = 8 from 4 independent experiments, two-sided *t*-test. **(d)** Representative images of GFP and GFP-SIRT1 WT or mutants in KECs subjected to shear stress for 1 day. NLS^{mut}: SIRT1 mutant retained in the cytosol. NES^{mut}: SIRT1 mutant retained in the nucleus. Scale bars, 10 µm. **(e,f)** Expression of *Ppargc1a* **(e)** and *Tfam* **(f)** in KECs subjected to flow (shear) or not (static) during 24h, in the presence or absence of EX527. mRNA levels were quantified by real-time RT-qPCR, normalized to β-actin and are presented as fold increases. Data show the mean ± s.e.m.; n = 3 independent experiments, two-sided *t*-test. Source data are provided as a Source Data file.

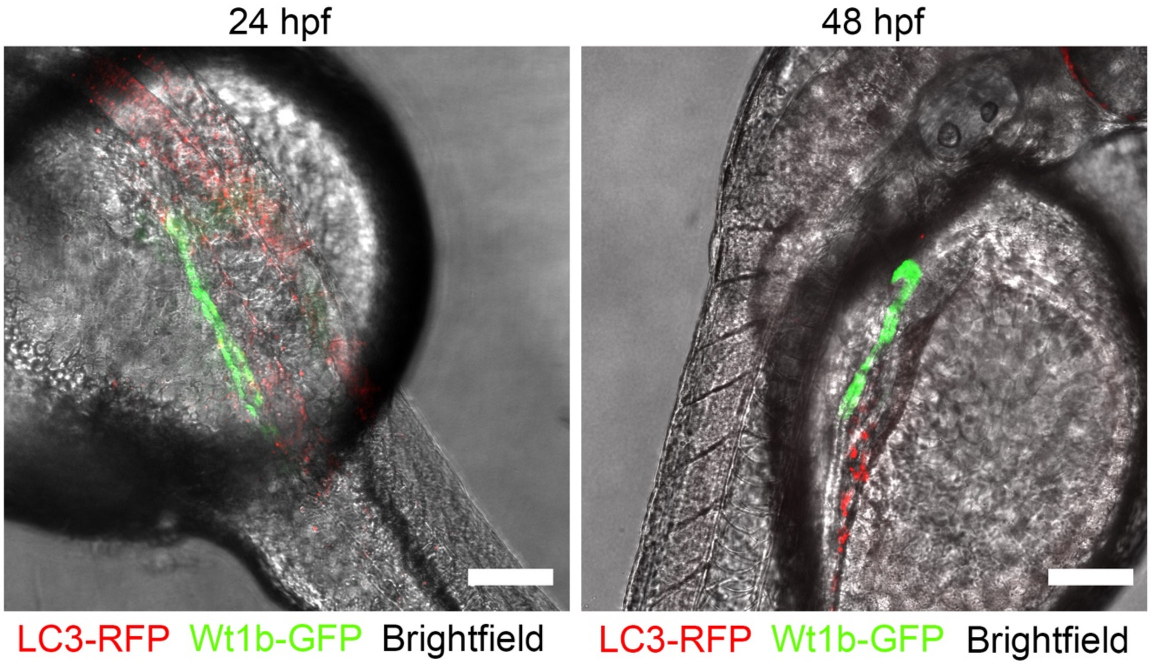

Supplementary Figure 8: Low magnification images of zebrafish pronephros.

Representative low magnification images of RFP-LC3 : Wt1b-GFP zebrafish pronephros with brightfield light, at 24h post fertilization (hpf) and 48 hpf. Scale bars, 100 µm. Source data are provided as a Source Data file.

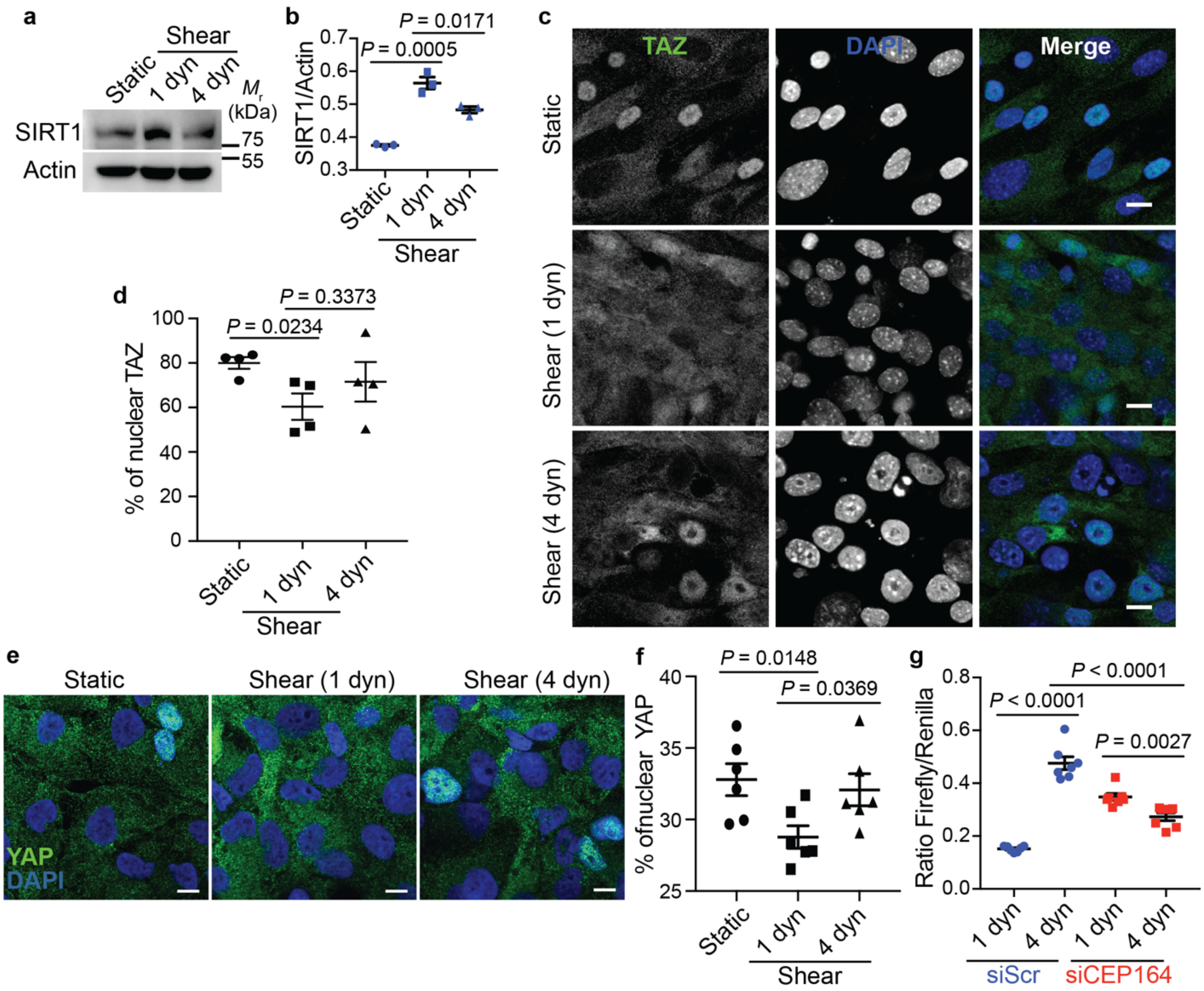

Supplementary Figure 9: Pathological flow does affect the expression of SIRT1 but does not induce TAZ nuclear translocation.

(a) Representative images of SIRT1 and Actin proteins levels in KECs subjected to physiological flow (shear 1 dyn) or not (static) during 48h. For pathological flow (4 dyn), KECs were subjected to physiological shear stress (1 dyn) during 24h before increasing the flow rate to 4 dyn.cm^{-2} during one more day. **(b)** The ratio of LC3-II to Actin was determined by densitometry, relative to panel a. Data show the mean ± s.e.m.; n = 3 independent experiments, two-sided *t*-test. **(c,d)** Representative images **(c)** and quantification **(d)** of TAZ nuclear localization in KECs subjected to physiological flow (shear 1 dyn) or not (static) during 48h. For pathological flow (4 dyn), KECs were

subjected to physiological shear stress (1 dyn) during 24h before increasing the flow rate to 4 $dyn.cm^{-2}$ during one more day. Data show the mean ± s.e.m.; n = 4 independent experiments, two-sided *t*-test. Scale bars, 10 µm. **(e,f)** Representative images **(e)** and quantification **(f)** of YAP nuclear localization in HK-2 subjected to physiological flow (shear 1 dyn) or not (static) during 48h. For pathological flow (4 dyn), HK-2 were subjected to physiological shear stress (1 dyn) during 24h before increasing the flow rate to 4 $dyn.cm^{-2}$ during one more day. Data show the mean ± s.e.m.; n = 4 independent experiments, two-sided *t*-test. Scale bars, 10 µm. **(g)** Luciferase assay for YAP/TAZ activity in KECs subjected or not (static) to physiological (1 dyn) or pathological (4 dyn) shear stress after a knockdown of CEP164. Data show the mean ± s.e.m.; n = 7 from 3 independent experiments, two-sided *t*-test. Source data are provided as a Source Data file.

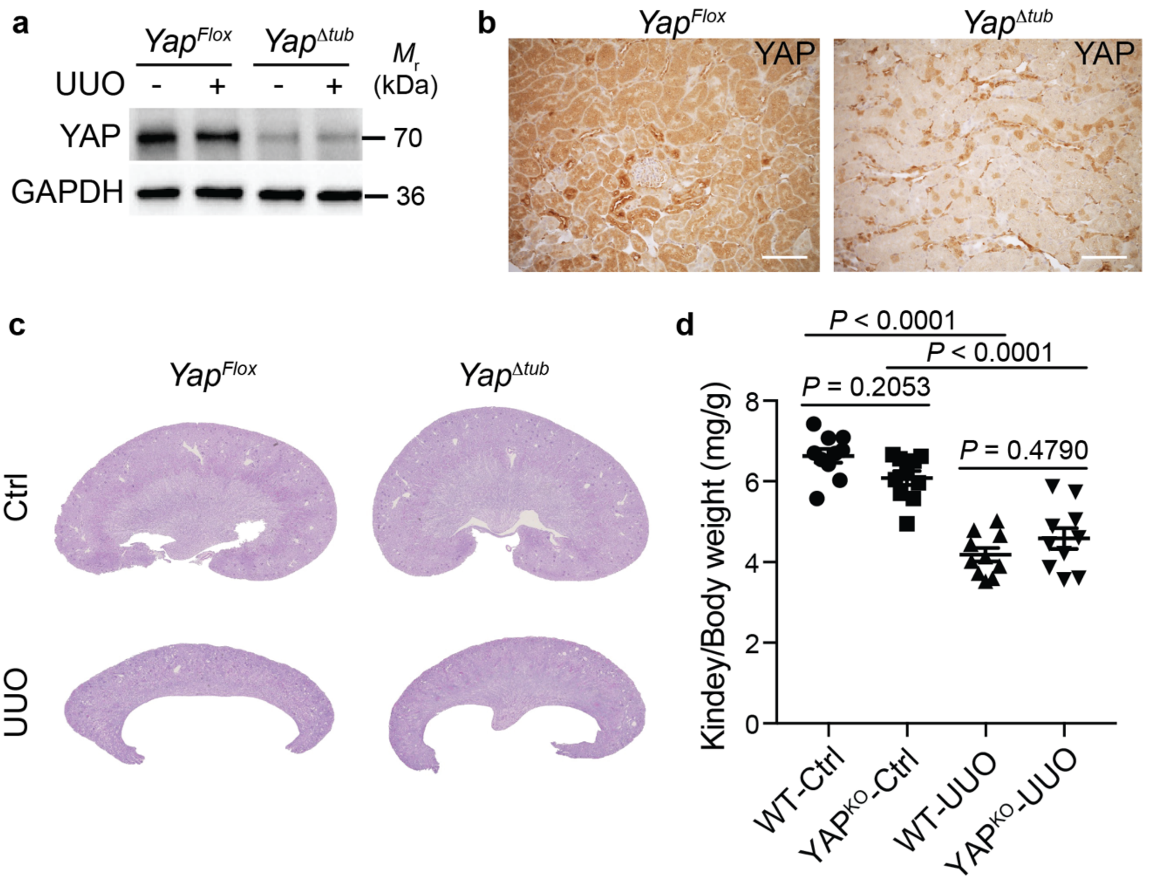

Supplementary Figure 10: The UUO and *Yap* tubular deletion murine models

(a) Representative images of YAP protein levels in $Yap^{\Delta tub}$ mice and Yap^{flox} littermates by western blot analysis on whole kidney extracts; n = 6 mice and **(b)** representative images of YAP expression in the kidney cortex of $Yap^{\Delta tub}$ mice and Yap^{flox} littermates by immunohistochemistry (original magnification 200X), n = 6 mice. **(c)** Representative images of the periodic acid-Schiff (PAS) stained control contralateral kidneys and obstructed kidneys (UUO (unilateral ureteral obstruction) kidney) 14 days after surgery in $Yap^{\Delta tub}$ mice and Yap^{flox} littermates (original magnification X25) and **(d)** kidney to-body weight ratio. Source data are provided as a Source Data file.

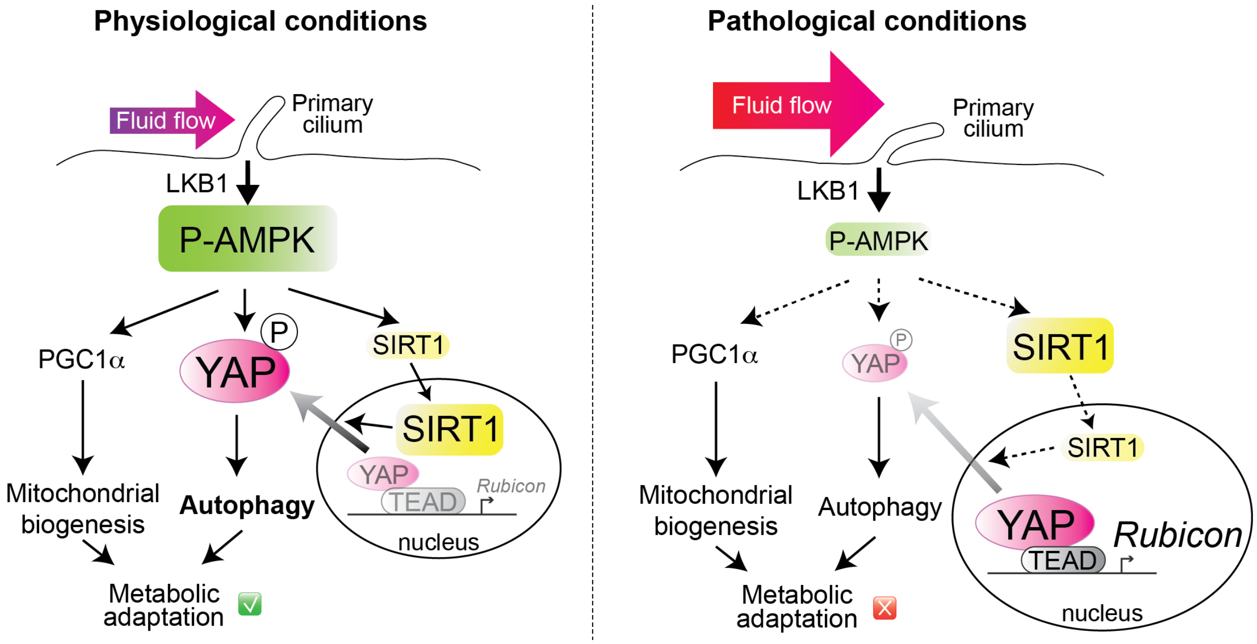

Supplementary Figure 11: Overall schematic summary.

In physiological conditions, the primary cilium-dependent activation of AMPK ensures the stimulation of 3 pathways: (i) SIRT1 activation is necessary to induce YAP exit from the nucleus (ii) AMPK-dependent phosphorylation of YAP on S61 sequestrate YAP in the cytosol, thus inhibiting the transcription of *Rubicon*, an autophagy suppressor. (iii) Increase of mitochondrial biogenesis. This metabolic reprogramming supports energy-consuming cellular processes such as glucose reabsorption and gluconeogenesis. In pathological conditions, the primary cilium-dependent activation of AMPK is impaired, resulting notably in the accumulation of nuclear YAP and inhibition of autophagy.
