# Supplementary material for: The AMPK-Sirtuin 1-YAP axis is regulated by fluid flow intensity and controls autophagy flux in kidney epithelial cells
Source: Nat Commun. 2023 Dec 5;14:8056. doi: 10.1038/s41467-023-43775-1 (PMC10698145; doi:10.1038/s41467-023-43775-1)
Supplement: Supplementary file 3 — Reporting Summary [file 41467_2023_43775_MOESM3_ESM.pdf]

nature portfolio

Corresponding author(s): Aurore Claude-Taupin and Nicolas Dupont

Last updated by author(s): Nov 3, 2023

Reporting Summary

Nature Portfolio wishes to improve the reproducibility of the work that we publish. This form provides structure for consistency and transparency in reporting. For further information on Nature Portfolio policies, see our Editorial Policies and the Editorial Policy Checklist.

Statistics

For all statistical analyses, confirm that the following items are present in the figure legend, table legend, main text, or Methods section.

n/a	Confirmed	
☐	☒	The exact sample size (*n*) for each experimental group/condition, given as a discrete number and unit of measurement
☐	☒	A statement on whether measurements were taken from distinct samples or whether the same sample was measured repeatedly
☐	☒	The statistical test(s) used AND whether they are one- or two-sided<br>*Only common tests should be described solely by name; describe more complex techniques in the Methods section.*
☐	☒	A description of all covariates tested
☒	☐	A description of any assumptions or corrections, such as tests of normality and adjustment for multiple comparisons
☐	☒	A full description of the statistical parameters including central tendency (e.g. means) or other basic estimates (e.g. regression coefficient) AND variation (e.g. standard deviation) or associated estimates of uncertainty (e.g. confidence intervals)
☐	☒	For null hypothesis testing, the test statistic (e.g. *F*, *t*, *r*) with confidence intervals, effect sizes, degrees of freedom and *P* value noted<br>*Give P values as exact values whenever suitable.*
☒	☐	For Bayesian analysis, information on the choice of priors and Markov chain Monte Carlo settings
☒	☐	For hierarchical and complex designs, identification of the appropriate level for tests and full reporting of outcomes
☒	☐	Estimates of effect sizes (e.g. Cohen's *d*, Pearson's *r*), indicating how they were calculated

Our web collection on statistics for biologists contains articles on many of the points above.

Software and code

Policy information about availability of computer code

Data collection	Zeiss Spinning disk piloted by Zen software 3.2, ChemiDoc MP Imaging System, Nanozoomer 2.0 HT (Hamamatsu), Pump control software v1.5.2 (Ibidi); qPCRsoft4.0 (qTOWER3G for mRNA relative expression, Analytik Jena); Design and Analysis Software 2.6.0 (QuantStudio 5 384 qPCR system for analysis of ChIP data, Thermo), ThermoTriStar2S LB942 (Berthold) piloted by MikroWin 2010 for luciferase assay.
Data analysis	ImageJ v2.1.0/1.53c with:<br>- Green and Red puncta colocalization macro (Showarski D.; Dagda R and Chu C available in GitHub: https://github.com/imagej/imagejdocu/blob/16d087e40ecc421a3812cddf8af01333572f9f8b/plugin/analysis/colocalization_analysis_macro_for_red_and_green_puncta/start.docu)<br>- Intensity Ratio Nuclei Cytoplasm Tool macro (Baecker V and Coux O available in GitHub: https://github.com/MontpellierRessourcesImagerie/imagej_macros_and_scripts.wiki.git)<br>- Mask creation with manual correction in 2D to quantify YAP intensity levels in nuclei and cytosol inside Zebrafish tubules. Macro designed by Nicolas Goudin, available upon request (nicolas.goudin@inserm.fr)<br>Icy (Licence GPLv3) with Spot Detector plugin v1.9.2.0 to quantify LC3 and Bodipy puncta (https://gitlab.pasteur.fr/bia/spot-detector) and the ROI statistics plugin to quantify cilia length using polyline 2D (https://gitlab.pasteur.fr/bia/roi-statistics).<br>Image Lab v5.2.1<br>Excel v16.16.23<br>Prism 9.5.0

For manuscripts utilizing custom algorithms or software that are central to the research but not yet described in published literature, software must be made available to editors and reviewers. We strongly encourage code deposition in a community repository (e.g. GitHub). See the Nature Portfolio guidelines for submitting code & software for further information.

Data

Policy information about availability of data

All manuscripts must include a data availability statement. This statement should provide the following information, where applicable:

- Accession codes, unique identifiers, or web links for publicly available datasets
- A description of any restrictions on data availability
- For clinical datasets or third party data, please ensure that the statement adheres to our policy

Authors confirm that all relevant data are included in the paper and/or its Supplementary Information files. Source Data for Figures 1-10 and Supplementary Figures 1-11 are provided with this paper. Resources and materials are available from Aurore Claude-Taupin (aurore.claude-taupin@inserm.fr) or Nicolas Dupont (nicolas.dupont@inserm.fr)

Research involving human participants, their data, or biological material

Policy information about studies with human participants or human data. See also policy information about sex, gender (identity/presentation), and sexual orientation and race, ethnicity and racism.

Reporting on sex and gender	Findings from this study do not apply to only one sex or gender and consent has been obtained for sharing of individual level data.
Reporting on race, ethnicity, or other socially relevant groupings	No socially constructed or socially relevant categorization variable has been used in our manuscript.
Population characteristics	We studied as controls 6 kidney biopsy samples from adult patients with normal kidney histology (minimal change disease) and 6 kidney biopsy samples from adult patients with moderate diabetic kidney disease. 50% of control kidneys samples were from male patients (n=3) and the rest from female patients (n=3). The mean age of the control population was of 45.8 with a standard deviation of 20.5. Regarding the DKD kidney samples, 66.7% were from male patients (n=4) and 33.3% from female patients (n=2). The mean age of the DKD population was of 57.3 with a standard deviation of 13.7.
Recruitment	Kidney and material from adult patients originated from our Pathology Department at Hôpital Necker-Enfants Malades (Paris, France). Our human kidney biopsy samples were taken in the context of patient care and then repositioned for biomedical research purposes. Written consent has been obtained from each patient, or their parents in the case of minors. No financial compensation is foreseen as these samples are taken in the context of care and not initially taken for research purposes.
Ethics oversight	The protocol was approved by the Institutional Review Board of Hôpital Necker-Enfants Malades, and informed written consent was obtained from all patients

Note that full information on the approval of the study protocol must also be provided in the manuscript.

Field-specific reporting

Please select the one below that is the best fit for your research. If you are not sure, read the appropriate sections before making your selection.

☒ Life sciences ☐ Behavioural & social sciences ☐ Ecological, evolutionary & environmental sciences

For a reference copy of the document with all sections, see nature.com/documents/nr-reporting-summary-flat.pdf

Life sciences study design

All studies must disclose on these points even when the disclosure is negative.

Sample size	No statistical method was used to predetermine sample size but these were instead determined based on our experiences from previous studies using similar methodologies. Experiments were independently repeated at least three times as indicated in the manuscript. Accordingly, the sample size was chosen based on previous experience with these experimental designs: Miceli et al., Nature Cell Biology 2020 https://doi.org/10.1038/s41556-020-0566-0; Boukhalfa et al. Nature Communications 2020 https://doi.org/10.1038/s41467-019-14086-1; Boukhalfa et al, Cell Reports 2021 https://doi.org/10.1016/j.celrep.2021.109045; Orhon et al, Nature Cell Biology 2016 https://doi.org/10.1038/ncb3360.
Data exclusions	No data were excluded for the analyses
Replication	Experiments were carried out in biological and/or technical replicates. The reproducibility of the experimental findings were verified by performing other independent experiments (at least two more).
Randomization	Microscopic images were acquired randomly. For cell-based experiments, cell lines were divided equally to each group and then treated with drug agents. No randomization of mice was done. They were age and sex-matched whenever possible. Zebrafish embryos were randomly selected based on their expression of LC3:RFP and Wt1b:GFP and equally divided in 24 hpf and 48 hpf groups.

Blinding

Investigators were blinded to group allocation for histology analyses. All other experiments were performed in non-blinded manner during data collection given the scale of experiments and readily observable differences between samples. Analysis using softwares minimized the occurrence of any bias.

Reporting for specific materials, systems and methods

We require information from authors about some types of materials, experimental systems and methods used in many studies. Here, indicate whether each material, system or method listed is relevant to your study. If you are not sure if a list item applies to your research, read the appropriate section before selecting a response.

Materials & experimental systems

n/a	Involved in the study	
☐	☒	Antibodies
☐	☒	Eukaryotic cell lines
☒	☐	Palaeontology and archaeology
☐	☒	Animals and other organisms
☒	☐	Clinical data
☒	☐	Dual use research of concern
☒	☐	Plants

Methods

n/a	Involved in the study	
☒	☐	ChIP-seq
☒	☐	Flow cytometry
☒	☐	MRI-based neuroimaging

Antibodies

Antibodies used

LC3B: Sigma, L7543,
LC3B: MBL, #M152.3,
LC3B: MBL, #PM036
Actin: Millipore, Clone C4, MAB1501,
LAMP1: Abcam, ab24170,
SQSTM1: PROGEN, GP62-C
ATG16L1: MBL, PM040,
WIPI2: Abcam, # ab105459;
YAP: Cell Signaling Technology, #14074,
YAP: Cell Signaling Technology, #4912,
Phospho-YAP_5127: Cell Signaling Technology, #13008,
Phospho-YAP_561: Cell Signaling Technology, #75784,
Phospho-YAP_5397: Cell Signaling Technology, #13619,
TAZ: Sigma, #HPA007415,
Phospho-TAZ_589: Cell Signaling Technology, #59971,
LATS1: Cell Signaling Technology, #9153,
Phospho-LATS1_T1079 : Cell Signaling Technology, #8654,
H3K9ac: Sigma, #07-352,
Histone H3: Proteintech, #17168-1-AP,
LKB1: Cell Signaling, #3050,
Acetylated lysine : Millipore, #06-933,
SIRT1: Millipore, #07-131,
FLAG-M2: Sigma, # F1804,
ARL13B: Proteintech, # 66739,
GFP : ab290, Abcam
gamma-tubulin: Sigma, #T5326,
Phospho-AMPK_T172: Cell Signaling Technology, #2535,
AMPK alphal: a gift from Grahame Hardie, University of Dundee, Dundee, UK.
Donkey anti-Mouse IgG (H+L) Highly Cross-Adsorbed Secondary Antibody, Alexa Fluor 647,A31571 - Invitrogen,
Donkey anti-Rabbit IgG (H+L) Highly Cross-Adsorbed Secondary Antibody, Alexa Fluor 647,A31573 — Invitrogen
Donkey anti-Mouse IgG (H+L) Highly Cross-Adsorbed Secondary Antibody, Alexa Fluor 488,A21202 — Invitrogen,
Donkey anti-Rabbit IgG (H+L) Highly Cross-Adsorbed Secondary Antibody, Alexa Fluor 488,A21206 — Invitrogen,
Donkey anti-Mouse IgG (H+L) Highly Cross-Adsorbed Secondary Antibody, Alexa Fluor 546,-A10036 — Invitrogen,
Goat anti-Rabbit IgG (H+L) Cross-Adsorbed Secondary Antibody, Alexa Fluor 555, A21428 — Invitrogen,
Donkey anti-Goat IgG (H+L) Cross-Adsorbed Secondary Antibody, Alexa Fluor" 488-nvitrogen,
HRP-labelled anti-rabbit : Millipore, #AP307P
HRP-labelled anti-mouse : Millipore, #AP308P
HRP-labelled anti-sheep : Calbiochem, #402100
Biotinylated anti-rabbit antibody (GE Healthcare, RPN1004V)

Validation

Goat anti-Rabbit IgG (H+L) Cross-Adsorbed Secondary Antibody, Alexa Fluor 555, A21428 — Invitrogen,
To minimize cross-reactivity, these goat anti-rabbit IgG (H+L) whole secondary antibodies have been affinity purified and crossadsorbed against human IgG, human serum, mouse IgG, mouse serum, and bovine serum. Cross-adsorption or pre-adsorption is a purification step to increase specificity of the antibody resulting in higher sensitivity and less background staining. Invitrogen'" Alexa Fluor 555 dye is a bright, orange-fluorescent dye with excitation ideally suited to the 555 nm laser line. For stable signal generation in

imaging and flow cytometry, Alexa Fluor 555 dye is pH-insensitive over a wide molar range.
This antibody has been cited by more than 1000 publications, as referenced by the manufacturer's website: https://www.thermofisher.com/antibody/product/Goat-anti-Rabbit IgG H L Cross-Adsorbed-Secondary-Antibody-Polyclonal/A-21428

Donkey anti-Mouse IgG (H+L) Highly Cross-Adsorbed Secondary Antibody, Alexa Fluor 546, A10036 — Invitrogen,
These donkey anti-mouse IgG whole secondary antibodies have been affinity-purified and show minimum cross-reactivity to bovine, chicken, goat, guinea pig, hamster, horse, human, rabbit, rat, and sheep serum proteins. Cross-adsorption or pre-adsorption is a purification step to increase specificity of the antibody resulting in higher sensitivity and less background staining. Invitrogen'" Alexa Fluor 546 dye is a bright, orange-fluorescent dye with excitation ideally suited to the 546 nm laser line. For stable signal generation in imaging and flow cytometry, Alexa Fluor 546 dye is pH-insensitive over a wide molar range.
This antibody has been cited by more than 380 publications, as referenced in the manufacturer's website, including:
FYCO1 and autophagy control the integrity of the haploid male germ cell-specific RNP granules. Da Ros M,Lehtiniemi T,Olotu O,Fischer D,Zhang FP,Vihinen H,Jokitalo E,Sironen A,Toppari J,Kotaja N, Autophagy 2, 302-321 (2017).

Donkey anti-Rabbit IgG (H+L) Highly Cross-Adsorbed Secondary Antibody, Alexa Fluor 488, A21206 — Invitrogen,
To minimize cross-reactivity, these donkey anti-rabbit IgG whole antibodies have been affinity-purified and show minimum crossreactivity to bovine, chicken, goat, guinea pig, hamster, horse, human, mouse, rat, and sheep serum proteins. Cross-adsorption or pre-adsorption is a purification step to increase specificity of the antibody resulting in higher sensitivity and less background staining. InvitrogenT" Alexa Fluor 488 dye is a bright, green-fluorescent dye with excitation ideally suited to the 488 nm laser line. For stable signal generation in imaging and flow cytometry, Alexa Fluor 488 dye is pH-insensitive over a wide molar range.
This antibody has been cited by more than 5,800 publications, as referenced in the manufacturer's website, including:
Tissue-engineered 3D human lymphatic microvascular network for in vitro studies of lymphangiogenesis. Gibot L,Galbraith T,Bourland J,Rogic A,Skobe M,Auger FA, Nature protocols 5, 1077-1088 (2017)

Donkey anti-Mouse IgG (H+L) Highly Cross-Adsorbed Secondary Antibody, Alexa Fluor 488, A21202 — Invitrogen
To minimize cross-reactivity, these donkey anti-mouse IgG whole antibodies have been affinity-purified and show minimum crossreactivity to bovine, chicken, goat, guinea pig, hamster, horse, human, mouse, rat, and sheep serum proteins. Cross-adsorption or pre-adsorption is a purification step to increase specificity of the antibody resulting in higher sensitivity and less background staining.
Invitrogen"" Alexa Fluor 488 dye is a bright, green-fluorescent dye with excitation ideally suited to the 488 nm laser line. For stable signal generation in imaging and flow cytometry, Alexa Fluor 488 dye is pH-insensitive over a wide molar range.
This antibody has been cited by more than 4,300 publications, as referenced in the manufacturer's website, including:
The Identification of Three Cancer Stem Cell Subpopulations within Moderately Differentiated Lip Squamous Cell Carcinoma. Ram R,Brasch HD,Dunne JC,Davis PF,Tan ST,Itinteang T, Frontiers in surgery, 4-12 (2017)

Donkey anti-Rabbit IgG (H+L) Highly Cross-Adsorbed Secondary Antibody, Alexa Fluor 647, A31573 — Invitrogen
To minimize cross-reactivity, these donkey anti-rabbit IgG whole antibodies have been affinity-purified and show a published crossreactivity to rat IgG. Cross-adsorption or pre-adsorption is a purification step to increase specificity of the antibody resulting in higher sensitivity and less background staining. nvitrogen" Alexa Fluor 647 dye is a near-infrared-fluorescent dye with excitation ideally suited to the 647 nm laser line. For stable signal generation in imaging and flow cytometry, Alexa Fluor 647 dye is pH-insensitive over a wide molar range.
This antibody has been cited by more than 1,900 publications, as referenced in the manufacturer's website, including:
Temporal Characterization of Microglia/Macrophage Phenotypes in a Mouse Model of Neonatal Hypoxic-Ischemic Brain Injury. Hellstrom Erkenstam N,Smith PL,Fleiss B,Nair S,Svedin P,Wang W,Bostrom M,Gressens P,Hagberg H,Brown KL,Savman K,Mallard C, Frontiers in cellular neuroscience 10-286 (2016)

Donkey anti-Mouse IgG (H+L) Highly Cross-Adsorbed Secondary Antibody, Alexa Fluor 647, A31571 - Invitrogen
To minimize cross-reactivity, these donkey anti-mouse IgG whole antibodies have been affinity-purified and show minimum crossreactivity to bovine, chicken, goat, guinea pig, hamster, horse, human, rabbit, rat, and sheep serum proteins. Cross-adsorption or pre-adsorption is a purification step to increase specificity of the antibody resulting in higher sensitivity and less background staining.
Invitrogen"" Alexa Fluor 647 dye is a near-infrared-fluorescent dye with excitation ideally suited to the 647 nm laser line. For stable signal generation in imaging and flow cytometry, Alexa Fluor 647 dye is pH-insensitive over a wide molar range.
This antibody has been cited by more than 1,600 publications, as referenced in the manufacturer's website, including:
Plasma Exosomes Spread and Cluster Around -Amyloid Plaques in an Animal Model of Alzheimer's Disease. Zheng T,Pu J,Chen Y,Mao Y,Guo Z,Pan H,Zhang L,Zhang H,Sun B,Zhang B, Frontiers in aging neuroscience, 9-12 (2017)

Donkey anti-Goat IgG (H+L) Cross-Adsorbed Secondary Antibody, Alexa Fluor" 488, A21206 -Invitrogen
Anti-Goat secondary antibodies are affinity-purified antibodies with well-characterized specificity for goat immunoglobulins and are useful in the detection, sorting or purification of its specified target. Secondary antibodies offer increased versatility enabling users to use many detection systems (e.g. HRP, AP, fluorescence). They can also provide greater sensitivity through signal amplification as multiple secondary antibodies can bind to a single primary antibody. Most commonly, secondary antibodies are generated by immunizing the host animal with a pooled population of immunoglobulins from the target species and can be further purified and modified (i.e. immunoaffinity chromatography, antibody fragmentation, label conjugation, etc.) to generate highly specific reagents.
This antibody has been cited by more than 1,600 publications, as referenced in the manufacturer's website:
https://www.thermofisher.com/antibody/product/Donkey-anti-Rabbit IgG H L Highly Cross-Adsorbed-Secondary-Antibody-Polyclonal/A-21206

HRP-labelled anti-rabbit : Millipore, #AP307P , Detect Rabbit IgG using this Goat anti-Rabbit IgG Antibody, (H+L) HRP conjugate validated for use in ELISA, IC, IH & WB., The reagent is an affinity purified antibody from goat. The purified antibody is conjugated to horseradish peroxidase (HRP) and stabilized in buffer. Specific for rabbit IgG, heavy and light chain. The cross-reactivities of antirabbit IgG antibody are tested in an ELISA. Minimum cross-reactivity to human IgG

HRP-labelled anti-mouse : Millipore, #AP308P, This Goat anti-Mouse IgG Antibody, (H+L) HRP conjugate is validated for use in ELISA, IC, IH, WB for the detection of Mouse IgG. This Goat anti-Mouse IgG Antibody, (H+L) HRP conjugate is validated for use in ELISA, IC, IH, WB for the detection of Mouse IgG. Specific for mouse IgG, heavy and light chain. The cross-reactivities of anti-mouse IgG

antibody are tested in an ELISA. Minimum cross-reactivity to human IgG

HRP-labelled anti-sheep: Calbiochem, #402100. This Rabbit Anti-Sheep IgG, H & L Chain Specific Peroxidase Conjugugate is validated for use in WB and has been previously used in: Alannan et al., Cells 2023 (PMID: 36611859).

Biotinylated anti-rabbit antibody (GE Healthcare, RPN1004V): source Donkey, The antibody is prepared by hyper-immunizing donkeys with purified immunoglobulin fractions from normal rabbit serum to produce high affinity antibodies. Biotin is attached to free amino groups on the purified antibodies via a spacer arm. Excess labelling reagent is removed by extensive dialysis.

LC3 (Sigma, L7543): Anti-LC3B antibody produced in rabbit has been used in western blotting. By immunoblotting, the antibody recognizes human, rat, and mouse LC3B-I and LC3B-II (- 18 kDa and —16 kDa, respectively). Validation: Functional assay. This antibody has been referenced by several studies, including: Rouschop KM, et.al. The unfolded protein response protects human tumor cells during hypoxia through regulation of the autophagy genes MAP1LC3B and ATGS. J Clin Invest 120, 127-141 (2010) or Swaminathan B, et.al. Autophagic marker MAP1LC3B expression levels are associated with carotid atherosclerosis symptomatology. PLoS ONE 9,e115176-e115176, (2014).

LC3B: MBL, #M152.3 :This antibody was purified from hybridoma (clone 4E12) supernatant using protein A agarose. This hybridoma was established by fusion of mouse myeloma cell P3U1 with C3H mouse lymphocyte immunized with the recombinant human LC3 [MAP1LC3B (1-120 aa)]. This antibody reacts with LC3 (MAP1LC3A, B) on Immunocytochemistry, Western blotting and Immunoprecipitation. REFERENCES:
1) Kabeya, Y., et al., J. Cell Sci. 117, 2805-2812 (2004) 2) Mizushima, N., et al., Mol. Biol. Cell 15, 1101-1111 (2004) 3) Mizushima, N., et al., J. Cell Biol. 152, 657-667 (2001) 4) Kabeya, Y., et al., EMBO J. 19, 5720-5728 (2000)

LC3B: MBL, #PM036: This antibody was purified from rabbit serum using protein A agarose. The rabbit was immunized with the recombinant human LC3 [MAP1LC3B (1-120 aa)].This antibody reacts with LC3 (MAP1LC3A, B, C). It does not react with GABARAP and GATE-16. Referenced in 1) Festa, B.P., et al., Nat. Commun. 9, 161 (2018) [WB] 2) Wang, Y., et al., Cell 171, 331-345.e22 (2017) [IC] 3) Meng,X.H.,etal.,Int.J.Biol.Sci.13,985-995(2017)[WB] 4) Fujita, N., et al., Elife 6, e23367 (2017) [Immuno-EM] 5) Botbol, Y., et al., Autophagy 11, 1864-1877 (2015) [IP] 6) Kaminskyy,V.O.,etal.,Autophagy8,1032-1044(2012)[IC] 7) Kaminskyy, V., et al., Autophagy 7, 83-90 (2011) [FCM] 8) Hasui, K., et al., Acta Histochem Cytochem 44, 119-131 (2011) [IHC] 9) Tabata, K., et al., Mol. Biol. Cell 21, 4162-4172 (2010) [WB] 10) Saitoh, T., et al., Nature 456, 264-268 (2008) [WB] 11) Wan,G.,etal.,J.Biol.Chem.283,21540-21549(2008) [WB] 12) Ohne,Y.,etal.,J.Biol.Chem.283,31861-31870(2008)[WB] 13) Kabeya, Y., et al., J. Cell Sci. 117, 2805-2812 (2004) 14) Mizushima,N.,etal.,MOl.Biol.Cell15,1101-1111(2004) 15) Mizushima, N., et al., J. Cell Biol. 152, 657-667 (2001) 16) Kabeya, Y., et al., EMBO J. 19, 5720-5728 (2000)

LC3B: MBL, #M152.3: This antibody was purified from hybridoma(clone 4E12) supernatant using protein A agarose. This hybridoma was established by fusion of mouse myeloma cell P3U1 with C3H mouse lymphocyte immunized with the recombinant human LC3 [MAP1LC3B (1-120 aa). This antibody reacts with LC3 (MAP1LC3A, B) on Immunocytochemistry, Western blotting and Immunoprecipitation. It has been referenced by several publications, including: 1) Kabeya, Y., et al., J. Cell Sci. 117, 2805-2812 (2004) 2) Mizushima, N., et al., Mol. Biol. Cell 15, 1101-1111 (2004); 3) Mizushima, N., et al., J. Cell Biol. 152, 657-667 (2001) and 4) Kabeya, Y., et al., EMBO J. 19, 5720-5728 (2000).

LAMP1: Abcam, ab24170„ Rabbit polyclonal to LAMP1 - Lysosome Marker. Immunogen= Synthetic peptide corresponding to Human LAMP1 aa 400 to the C-terminus (C terminal) conjugated to keyhole limpet haemocyanin. This antibody has been referenced by more than 650 studies according to the manufacturer's website: https://www.abcam.com/products/primary-antibodies/lamp1-antibodylysosome-marker-ab24170.html

SQSTM1: PROGEN, GP62-C, Guinea pig polyclonal antibody Suitable for IHC and WB, Reacts with bovine, human, mouse and rat, Immunogen C-terminal domain (20 amino acids: C-NYD IGA ALD TIQYSK HPP PL) of human p62 protein, coupled to KLH. This peptide sequence is identical in human, monkey, bovine, mouse, and rat. This antibody has been used in more than 240 publications, according to the manufacturer's website: https://www.progen.com/anti-p62-SQSTM1-C-terminus-guinea-pig-polyclonal-serum/GP62-C.

Actin: Reliably and specifically detect actin using this Anti-Actin Antibody, clone C4. This highly published monoclonal antibody is validated for use in ELISA, IC, IF, IH, IH(P) & WB. This mAb is also available as a fluorescent conjugate. Purified chicken gizzard actin (Lessard, 1988). Elevated expression of mechanosensory polycystins in human carotid atherosclerotic plaques: association with p53 activation and disease severity. Referenced in Choudhury S et al., Caspase-7: a critical mediator of optic nerve injury-induced retinal ganglion cell death, Scientific reports 2015.

ATG16L1, MBL, PM040,: This antibody reacts with Atg16L on Western blotting, Immunoprecipitation and Immunocytochemistry. It is produced in rabbit. Referenced in Matsunaga K et al. Autophagy requires endoplasmic reticulum targeting of the P13-kinase complex via Atg14L. J Cell Biol. 190, 511-21 (2010) and Moreau K et al. Arf6 promotes autophagosome formation via effects on phosphatidylinositol 4,5-bisphosphate and phospholipase D. J Cell Biol. 196, 483-96 (2012).

WIPI2: Abcam, # ab105459, Mouse monoclonal [2A2] to WIPI2, Immmunogen= Synthetic peptide corresponding to Human WIPI2 aa 400-500 (C terminal). Referenced by more than 50 studies, according to the manufacturer's website: https://www.abcam.com/products/primary-antibodies/wipi2-antibody-2a2-ab105459.html

YAP: Cell Signaling Technology, #14074, YAP (D8H1X) XP® Rabbit mAb recognizes endogenous levels of total YAP protein. Species Reactivity: Human, Mouse, Rat, Hamster, Monkey. Monoclonal antibody is produced by immunizing animals with recombinant protein specific to the carboxy terminus of human YAP protein. The epitope corresponds to a region surrounding Pro435 of human YAP isoform 1. This sequence region is 100% conserved among all known isoforms of human YAP protein. This antibody has been referenced by more than 700 publications, according to the manufacturer's website: https://www.cellsignal.com/products/primaryantibodies/yap-d8h1x-xp-rabbit-mab/14074?_=1686228564915&Ntt=14074&tahead=true

YAP: Cell Signaling Technology, #4912, YAP Antibody detects endogenous levels of total YAP protein. Polyclonal antibodies are produced by immunizing animals with a synthetic peptide corresponding to residues surrounding His104 of human YAP protein. Antibodies are purified by protein A and peptide affinity chromatography. This antibody has been referenced by more than 500 studies, according to the manufacturer's website: https://www.cellsignal.com/products/primary-antibodies/yap-antibody/4912

Phospho-YAP_S127: Cell Signaling Technology, #13008: Phospho-YAP (Ser127) (D9W21) Rabbit mAb recognizes endogenous levels of YAP protein only when phosphorylated at Ser127. This antibody may cross-react with phospho-TAZ (Ser89). Source / Purification=Monoclonal antibody is produced by immunizing animals with a synthetic phosphopeptide corresponding to residues surrounding Ser127 of human YAP protein. This antibody has been referenced by more than 300 studies, according to the manufacturer's website: https://www.cellsignal.com/products/primary-antibodies/phospho-yap-ser127-d9w2i-rabbit-mab/13008

Phospho-YAP_561: Cell Signaling Technology, #75784. Phospho-YAP (Ser61) Antibody recognizes endogenous levels of YAP protein only when phosphorylated at Ser61. This antibody does not cross-react with phosphorylated TAZ, due to the absence of an equivalent modification site in the TAZ protein.Source / Purification= Polyclonal antibodies are produced by immunizing animals with a synthetic phosphopeptide corresponding to residues surrounding Ser61 of human YAP protein. Antibodies are purified by peptide affinity chromatography.

Phospho-YAP_S397: Cell Signaling Technology, #13619, Phospho-YAP (Ser397) Rabbit mAb recognizes endogenous levels of YAP protein only when phosphorylated at Ser397. This residue corresponds to Ser381 of YAP isoform 2, as reported by Zhao, B. et al. (2010) Genes Dev 24, 72-85 (9), Source / Purification: Monoclonal antibody is produced by immunizing animals with a synthetic phosphopeptide corresponding to residues surrounding Ser397 of human YAP protein isoform 1. This antibody has been referenced by more than 60 studies, according to the manufacturer's website: https://www.cellsignal.com/products/primary-antibodies/phospho-yap-ser397-d1e7y-rabbit-mab/13619

TAZ: Sigma, #HPA007415, polyclonal rabbit affinity isolated antibody, Immunogene: WW domain-containing transcription regulator protein 1 recombinant protein epitope signature tag (PrEST). This antibody has been validated for IHC, WB and IF in cell lines: https://www.proteinatlas.org/ENSG00000018408-WVVTR1/subcellular.

Phospho-TAZ_589: Cell Signaling Technology, #59971 (E1X9C) Rabbit mAb recognizes endogenous levels of TAZ protein only when phosphorylated at Ser89. Due to sequence similarities near the phosphorylation site, this antibody may also detect endogenous levels of YAP protein when phosphorylated at Ser127, Source / Purification:Monoclonal antibody is produced by immunizing animals with a synthetic phosphopeptide corresponding to residues surrounding Ser89 of human TAZ protein. This antibody has been referenced by more than 40 studies, according to the manufacturer's website: https://www.cellsignal.com/products/
primaryantibodies/
phospho-taz-ser89-e1x9c-rabbit-mab/59971

LATS1: Cell Signaling Technology, #9153: LATS1 Antibody detects endogenous levels of total LATS1 protein.Species Reactivity: Human, Monkey. Source / Purification: Polyclonal antibodies are produced by immunizing animals with a synthetic peptide corresponding to amino acids surrounding Ser177 of human LATS1. Antibodies are purified by protein A and peptide affinity chromatography. This antibody has been referenced by more than 60 studies, according to the manufacturer's website: https://www.cellsignal.com/products/primary-antibodies/lats1-antibody/9153

Phospho-LATS1_T1079 : Cell Signaling Technology, #8654: Phospho-LATS1 (Thr1079) (D57D3) Rabbit mAb detects endogenous levels of LATS1 protein only when phosphorylated at Thr1079. This antibody is predicted to cross react with LATS2 only when LATS2 is phosphorylated at Thr1041. Source / Purification: Monoclonal antibody is produced by immunizing animals with a synthetic phosphopeptide corresponding to residues surrounding Thr1079 of human LATS1 protein. This antibody has been referenced by more than 170 studies, according to the manufacturer's website: https://www.cellsignal.com/products/primary-antibodies/phospholats1-thr1079-d57d3-rabbit-mab/8654

H3K9ac: Sigma, #07-352, Recognizes acetyl-histone H3 (Lys9), Mr 17 kDa. Anti-acetyl-Histone H3 (Lys9) Antibody is a Rabbit Polyclonal Antibody for detection of acetyl-Histone H3 (Lys9) also known as H3K9Ac, Histone H3 (acetyl K9) and has been published and validated in ChIP, WB, Mplex. Epitope: a.a. 4-14 Ovalbumin-conjugated, synthetic peptide (KQTARAcKSTGGK-C) corresponding to amino acids 4-14 of yeast histone H3 acetylated on lysine 9, with a C-terminal cysteine added for conjugation purposes. This antibody has been referenced in more than 500 studies, according to the manufacturer's website: https://www.merckmillipore.com/FR/fr/product/Anti-acetyl-Histone-H3-Lys9-Antibody,MM_NF-07-352?ReferrerURL=https%3A%2F%2Fwww.google.com%2F&bd=1#anchor_REF

Histone H3 (Proteintech,17168-1-AP) targets Histone-H3 in WB, IP, IHC, IF, FC, CoIP, ChIP, ELISA applications and shows reactivity with human, mouse, rat samples. Polyclonal Rabbit antobdy. Purification Method Antigen affinity purification. This antibody has been referenced in more than 670 studies, according to the manufacturer's website: https://www.ptglab.com/products/Histone-H3-Antibody-17168-1-AP.htm

LKB1 (Cell Signaling, #3050, 27D10) Rabbit mAb detects endogenous levels of LKB1 protein. This Monoclonal antibody is produced by immunizing animals with a synthetic peptide corresponding to the sequence of human LKB1. This antibody has been referenced in more than 100 studies, according to the manufacturer's website: https://www.cellsignal.com/products/primary-antibodies/lkb1-27d10-rabbit-mab/3050

Acetylated lysine : Millipore, #06-933, Anti-acetyl-Lysine Antibody is a high quality Rabbit Polyclonal Antibody for the detection of acetyl-Lysine & has been validated in WB & IP and many studies, including: Mitchell Let al., mChIP-KAT-MS, a method to map protein interactions and acetylation sites for lysine acetyltransferases, PNAS 2013.

SIRT1: Millipore, #07-131: Anti-Sirt1(Sir2), Cat. No. 07-131, is a rabbit polyclonal antibody that detects Sirt1/Sir2 and is tested for use in Chromatin Immunoprecipitation (ChIP), Immunocytochemistry, and Western Blotting. This antibody has been cited in more than 250 publications, as referenced in the manufacturer's website: https://www.merckmillipore.com/FR/fr/product/Anti-Sirt1Sir2-Antibody,MM_NF-07-131?ReferrerURL=https%3A%2F%2Fwww.google.com%2F&bd=1

FLAG-M2: Sigma, # F1804, M2, monoclonal, The ANTI-FLAG M2 mouse, affinity purified monoclonal antibody binds to fusion proteins containing a FLAG peptide sequence.The antibody recognizes the FLAG peptide sequence at the N-terminus, Met-Nterminus,C-terminus, and internal sites of the fusion protein, Immmmunogene: DYKDDDDK. This antibody has been referenced in more than 8,000 publications, including Diaz-Ortiz M et al., GPNMB confers risk for Parkinson's disease through interaction with -synuclein. Science 2022

ARL13B: Proteintech, # 66739, ARL13B Monoclonal Antibody (CloneNo.1H6C3) for IF, WB, ELISA and shows reactivity with Canine, Human, mouse, pig samples. This antibody has been referenced by more than 10 publications, as referenced by the supplier's website: https://www.ptglab.com/products/ARL13B-Antibody-66739-1-Ig.htm

GFP : ab290, Abcam, Rabbit polyclonal to GFP, Immunogen: Recombinant full length protein corresponding to GFP. Green fluorescent protein (GFP) from Aequorea victoria. This antibody has been referenced by more than 2,600 publications, as referenced by the supplier's website: https://www.abcam.com/products/primary-antibodies/gfp-antibody-ab290.html

gamma-tubulin: Sigma, #T5326, The antibody recognizes an epitope located within the N-terminal region of -tubulin. Immmmunogene: synthetic -tubulin peptide, conjugated to KLH. This antibody has been referenced by more than 390 publications, including Solier Set al., A druggable copper-signalling pathway that drives inflammation, Nature 2023.

AMPK alpha1: a gift from Grahame Hardie, University of Dundee, Dundee, UK. This antibody was purified in sheep and validated by the use of AMPKalphal-deficient mice as well as siRNA directed targeted. This antibody has been previously used and validated in Lantier et al., AMPK controls exercise endurance, mitochondrial oxidative capacity, and skeletal muscle integrity FASEB J 2014.

Phospho-AMPK_T172: Cell Signaling Technology, #2535 (40H9) Rabbit mAb detects endogenous AMPK 1 only when phosphorylated at threonine 183 and endogenous AMPK 2 only when phosphorylated at threonine 172. The antibody does not detect the regulatory or subunits. Source / Purification: Monoclonal antibody is produced by immunizing animals with a synthetic peptide corresponding to residues surrounding Thr172 of human AMPK 2 protein. This antibody has been referenced by more than 3,200 publications, as referenced by the supplier's website: https://www.cellsignal.com/products/primary-antibodies/phospho-ampkathr172-40h9-rabbit-mab/2535

Eukaryotic cell lines

Policy information about cell lines and Sex and Gender in Research

Cell line source(s)	Kidney mouse epithelial cells (KECs) generated by GJ Pazour (University of Massachusetts, Worcester, MA, USA) were kindly provided by A.M. Cuervo (Albert Eisntein College, Bronx, NY, USA). HK-2 cells were obtained from American Type Culture Collection (ATCC). CRISPR Yap-knockout cells were generated in this work.
Authentication	The cell lines were not authenticated.
Mycoplasma contamination	The cell lines are routinely tested negative for mycoplasma.
Commonly misidentified lines (See ICLAC register)	No commonly misidentified cell lines were used in the study.

Animals and other research organisms

Policy information about studies involving animals; ARRIVE guidelines recommended for reporting animal research, and Sex and Gender in Research

Laboratory animals	Mice were on a C57BL/6 (for Figures 5 and 7) or FVB/N (for Figure 10 and Sup Figure 10) genetic background (Janvier Laboratories or Jackson laboratory for Ella-Cre mice). Animals were housed in a specific pathogen-free facility, fed ad libitum (standard diet; 18% proteins, 6% fat, #2018, Inotiv produced from Mucedola, Milan, Italy) and housed at constant ambient temperature (between 20-21° C) in a 12-hour light cycle. For Figure 7, female mice were subjected to either unilateral ureteral obstruction or sham operation (at 8 week old) and sacrified 24 hours after surgery. For Figure 10 and Sup Figure 10, female mice were subjected to unilateral ureteral obstruction and sacrificed 14 days after surgery. At the time of sacrifice, the right control contralateral kidney and the left obstructed kidney were removed.<br><br>Zebrafish embryos (24 and 28 hpf) were obtained by natural mating of Tg(hsp70l:RFP-Rno.Map1lc3b) line (males, RFP-LC3) with the Tg(wt1b:GFP) line (females) and raised at 28.5°C in petri dishes containing fish water. As the experiments performed for this study were using zebrafish embryos less than 120 hpf, no ethical permission was needed.
Wild animals	The study did not involve wild animals.
Reporting on sex	Only female mice were used in this study.
Field-collected samples	The study did not involve samples collected in the field.
Ethics oversight	Animal procedures were approved by the Departmental Director of “Services Vétérinaires de la Préfecture de Police de Paris” and by the ethical committee of the Paris Cité University

Note that full information on the approval of the study protocol must also be provided in the manuscript.
